# Extracellular vesicles derived from LPS-preconditioned human synovial mesenchymal stem cells inhibit extracellular matrix degradation and prevent osteoarthritis of the knee in a mouse model

**DOI:** 10.1186/s13287-021-02507-2

**Published:** 2021-07-28

**Authors:** Ao Duan, Kai Shen, Beichen Li, Cong Li, Hao Zhou, Renyi Kong, Yuqi Shao, Jian Qin, Tangbo Yuan, Juan Ji, Wei Guo, Xipeng Wang, Tengfei Xue, Lei Li, Xinxin Huang, Yuqin Sun, Zhenyu Cai, Wei Liu, Feng Liu

**Affiliations:** 1 Department of Orthopedics, The First Affiliated Hospital of Nanjing Medical University, Nanjing, 210029 Jiangsu China; 2 Department of Orthopedics, Nanjing First Hospital, Nanjing Medical University, Nanjing, 210001 Jiangsu China; 3 Department of Orthopedics, Xincheng Hospital of Traditional Chinese Medicine, Maanshan, 243131 Anhui China; 4 Department of Orthopedics, Sir Run Run Hospital, Nanjing Medical University, Nanjing, 211100 China; 5 Department of Pharmacology, Neuroprotective Drug Discovery Key Laboratory, Jiangsu Key Laboratory of Neurodegeneration, Center for Global Health, Nanjing Medical University, Nanjing, 211100 China; 6 Department of Radiology, The First Affiliated Hospital of Nanjing Medical University, Nanjing, 210029 Jiangsu China

**Keywords:** Osteoarthritis, Extracellular vesicles, LPS-precondition, Let-7b, ADAMTS5

## Abstract

**Background:**

Previous studies report that lipopolysaccharide (LPS)-preconditioned mesenchymal stem cells have enhanced trophic support and improved regenerative and repair properties. Extracellular vesicles secreted by synovial mesenchymal stem cells (EVs) can reduce cartilage damage caused by osteoarthritis (OA). Previous studies show that extracellular vesicles secreted by LPS-preconditioned synovial mesenchymal stem cells (LPS-pre EVs) can improve the response to treatment of osteoarthritis (OA). This study sought to explore effects of LPS-pre EVs on chondrocyte proliferation, migration, and chondrocyte apoptosis, as well as the protective effect of LPS-pre EVs on mouse articular cartilage.

**Methods:**

Chondrocytes were extracted to explore the effect of LPS-pre EVs on proliferation, migration, and apoptosis of chondrocytes. In addition, the effect of LPS-pre EVs on expression level of important proteins of chondrocytes was explored suing in vitro experiments. Further, intraarticular injection of LPS-pre EVs was performed on the destabilization of the medial meniscus (DMM)-induced mouse models of OA to explore the therapeutic effect of LPS-pre EVs on osteoarthritis in vivo.

**Results:**

Analysis showed that LPS-pre EVs significantly promoted proliferation and migration of chondrocytes and inhibited the apoptosis of chondrocytes compared with PBS and EVs. Moreover, LPS-pre EVs inhibited decrease of aggrecan and COL2A1 and increase of ADAMTS5 caused by IL-1β through let-7b. Furthermore, LPS-pre EVs significantly prevented development of OA in DMM-induced mouse models of OA.

**Conclusions:**

LPS pretreatment is an effective and promising method to improve therapeutic effect of extracellular vesicles secreted from SMSCs on OA.

## Background

Osteoarthritis (OA) is a chronic degenerative joint disease characterized by degeneration of articular cartilage. It is associated with signs such as subchondral remodeling, osteophyte formation, and synovial inflammation [1]. Several factors are associated with pathogenesis of OA, such as age [2], joint injury [3], hip dysplasia [4, 5], femoroacetabular impingement morphology [6, 7], varus (or valgus) knee alignment [8, 9], and leg length inequality [10]. Age and joint injury are the most common factors leading to OA [2, 3]. Metabolic disorder of extracellular matrix (ECM) plays an important role in progression of OA. Collagen type II alpha 1 (COL2A1) and aggrecan are important components of ECM, and their abnormal synthesis and secretion results irreversible damage of ECM in cartilage [11, 12].

Most treatment approaches for OA can only relieve joint pain but do not repair joint injury [13]. In addition, current surgical treatments are limited by the severe trauma to the body and the finite lifespan of the prostheses [14]. Therefore, it is important to prevent or reverse progression of early osteoarthritis before it is advanced.

Recent studies report that stem cell transplantation has good therapeutic effects in treatment of degenerative diseases [15, 16]. Previous studies extracting mesenchymal stem cells (MSCs) from various tissues and applying them in tissue repair have significantly increased [17]. Animal and preclinical studies show that MSCs have great potential for cartilage regeneration [18, 19]. Synovial mesenchymal stem cells (SMSCs) were isolated from synovial membrane surrounding joints for the first time in 2001 [20]. SMSCs have great potential for cartilage regeneration research due to the tissue specificity of cartilage regeneration [21]. Moreover, SMSCs have the advantages of easy isolation, high proliferation ability, and slow aging compared with other MSCs [22]. Previous studies report that SMSCs retain the multi-lineage differentiation even after the tenth passage [22]. However, direct use of stem cells is partially limited by risk factors such as possible chromosomal variations and immune rejection [23, 24]. Therefore, studies should develop a superior method for effective use of stem cells and avoid possible risk related to direct utilization.

Extracellular vesicles are membrane vesicles with a diameter ranging between 50 and 200 nm that enter the cell matrix after integration of intracellular vesicles and cell membrane [25]. Extracellular vesicles may be critical messengers in cell-cell communication [26]. Most cells in the body can secrete extracellular vesicles, and extracellular vesicles have similar biological functions as their source cells. Direct use of these nanoparticles has no significant side effects, such as immunogenicity or tumorigenicity [27, 28]. Several studies report that efficacy of MSC-based therapy on cartilage regeneration is attributed to the paracrine activity of extracellular vesicles, which deliver specific substances to recipient cells [26, 29, 30]. Extracellular vesicles contain many functional microRNAs (miRNAs), which play important roles in the treatment of OA [1, 22, 31, 32]. Previous clinical studies report that EVs show close association with OA. They play roles in enhancing chondrocyte regeneration, suppressing apoptosis, and promoting ECM balance and thus play important roles in treatment of OA [22, 32, 33].

Studies report that combination of MSCs and lipopolysaccharide (LPS) can enhance the nutritional role and functional properties of this treatment, and exert resistance to harsh extracellular environments [34–36]. Further, pre-treating MSCs with low concentrations of the two non-toxic components before transplantation enhances tissue damage tolerance in different organs [37–40]. Moreover, extracellular vesicles secreted by LPS-preconditioned MSCs promote transformation of macrophages to M2-type and promote skin wound healing [41]. Currently, no studies have explored application of extracellular vesicles derived from LPS-pre EVs in OA treatment. This study postulated that LPS-pre EVs may be more effective in treatment of OA compared with EVs.

The findings of this study showed that LPS-pre-EVs significantly enhanced proliferation and migration of chondrocytes, inhibited apoptosis of chondrocytes, and protected of ECM from degradation compared with EVs. miRNA sequencing showed that LPS-pre EVs contained higher levels of let-7b compared with EVs. Let-7b targeted mRNA of the A disintegrin-like and metalloproteinase domain with thrombospondin-1 motifs 5 (ADAMTS5) and reduced expression of ADAMTS5. ADAMTS5 played a significant role in hydrolysis of ECM; thus, ECM in the LPS-pre EVs treatment group was effectively protected from degradation caused by OA. This study provides a new strategy for clinical treatment of OA by exploring functions of miRNAs in LPS-pre EVs.

## Methods

### Isolation and incubation of human chondrocytes

Knee cartilages were obtained from cases receiving total knee arthroplasty (THA). The study protocol was approved by the research ethics committee of the First Affiliated Hospital of Nanjing Medical University. Informed consent was obtained from OA patients with no additional systemic disorder. The cartilage tissue was washed three times with phosphate buffer saline (PBS) containing 1% penicillin–streptomycin (Gibco). The washed cartilage tissue was then cut using a surgical blade into cubes with a rib length of 1 mm. Chondrocytes were first digested with PBS containing 2% trypsin (Gibco) for 30 min, then with complete medium containing 2 mg/ml collagenase type II (Gibco) for 12–16 h. Chondrocytes were then filtered through a cell sieve and centrifuged three times to obtain primary chondrocytes. Chondrocytes were cultured in Dulbecco’s Modified Eagle Medium/Nutrient Mixture F-12 (DMEM/F12, Gibco) containing 20% fetal bovine serum (FBS) and 1% penicillin–streptomycin (Gibco). IL-1β was added into the culture medium making a final concentration of 10 ng/ml to simulate the survival environment of chondrocytes in the state of OA as described previously [31]. Morphological characteristics of the cells were observed under an inverted microscope. Cells were placed in a cell incubator at a constant temperature of 37 °C and 5% CO_2_ for subsequent experiments.

### Isolation and incubation of SMSCs

Knee cartilage synovial tissues were obtained from THA cases. The synovial tissue was first washed three times with PBS containing 1% penicillin–streptomycin (Gibco). The adipose tissue was then removed, and the synovial tissue cut into small pieces using a pair of scissors. The synovial tissue sections were digested with complete medium containing 2 mg/ml collagenase type II (Gibco) for 12–16 h. After digestion, cells were filtered and centrifuged three times, and then cultured in a low-glucose DMEM (Gibco) containing 10% FBS and 1% penicillin–streptomycin. After 12 h of culture, the culture medium was changed to obtain the primary SMSCs, and cells at the fourth to fifth passage were used for subsequent experiments.

In both preconditioning, SMSCs (1.5 × 10^6^) were inoculated in a 15-cm culture dish for 24 h until a 70–80% cell confluence was attained. Cells were washed three times with PBS after aspiration of the medium. Cells were cultured with serum-free medium (Procell) alone (negative control) or medium containing 100 ng/ml LPS (Sigma). Cells then incubated for 48 h before collecting the supernatants.

### Preparation and identification of extracellular vesicles

Extracellular vesicles were obtained from LPS-pre MSCs derived supernatants as described previously [42, 43]. The supernatants were filtered with a 0.22-μm filter to remove dead cells and large fragments, followed by centrifugation 30 min at 10,000*g* to remove tiny fragments and centrifugation for 3 h at 140,000*g* to obtain extracellular vesicles were suspended in PBS for subsequent experiments.

EVs and LPS-pre EVs diameter distributions were analyzed using NanoSight LM10 system (NanoSight Ltd, Novato, CA). Transmission electron microscope (TEM; Tecnai 12; Philips, Best, The Netherlands) was used to assess morphology of extracellular vesicles. Western blot assay was performed to detect the specific surface markers of extracellular vesicles, such as CD9, CD63, Alix, and TSG101.

### Internalization of EVs and LPS-pre EVs into chondrocytes

Dil dye is a lipophilic fluorescent dye used to stain cell membranes and other lipid soluble biological structures. Therefore, it is used to stain extracellular vesicles [44]. To determine whether the chondrocytes could internalize extracellular vesicles, extracted extracellular vesicles were first labeled with 4 mg/ml Dil dye (Sigma) and then centrifuged at 140,000*g* for 1 h. Labeled extracellular vesicles were co-cultured with chondrocytes at 10^10^ particle/ml for 24 h. The nuclei were then stained with 4′,6-diamidino-2-phenylindole (DAPI, Thermo) for 15 min, and then observed and photographed using fluorescence microscope (Zeiss, Germany).

### Proliferation analysis of chondrocytes

Chondrocytes were divided into four groups including (1) control, (2) IL-1β + PBS, (3) IL-1β + EVs, and (4) IL-1β + LPS-pre EVs groups. Chondrocytes were seeded into 24-well plates and after chondrocytes of each group reached 70–80% confluence, IL-1β + PBS, IL-1β + EVs, and IL-1β + LPS-pre EVs groups were cultured with IL-1β (10 ng/ml) (Proteintech)-containing medium for 12 h. The medium for the control group was replaced with complete culture medium containing EdU (10 μM). The medium of the IL-1β + PBS group was replaced with complete culture medium containing IL-1β (10 ng/ml), PBS (100 μl), and EdU (10 μM). The medium for third group was replaced with complete culture medium containing IL-1β (10 ng/ml), EVs (10^10^ particles/ml), and EdU (10 μM), and the medium of the IL-1β + LPS-pre EVs group was replaced with complete culture medium containing IL-1β (10 ng/ml), LPS-pre EVs (10^10^ particles/ml), and EdU (10 μM). Extracellular vesicles-free serum was used in all the above complete media. Cells were incubated for 10 h. After incubation, analysis was conducted using BeyoClick™EdU-594 Cell Proliferation Detection Kit (Beyotime) following the manufacturer’s instructions. The nuclei of the proliferating cells emitted red fluorescence under excitation light of the fluorescence microscope. Cell proliferation of each group was observed and recorded using fluorescence microscope.

### Apoptosis analysis of chondrocytes

Chondrocytes were divided into four groups including (1) control group, (2) IL-1β + PBS group, (3) IL-1β + EVs group, and (4) IL-1β + LPS-pre EVs group. Chondrocytes of each group were seeded into 24-well plates, and after reaching 80–90% confluence, the medium for the control group was replaced with complete culture medium. The medium of the IL-1β + PBS group was replaced with complete culture medium containing IL-1β (10 ng/ml) and PBS (100ul). The medium for IL-1β + EVs group was replaced with complete culture medium containing IL-1β (10 ng/ml) and EVs (10^10^ particles/ml), whereas the medium for IL-1β + LPS-pre EVs group was replaced with complete culture medium containing IL-1β (10 ng/ml) and LPS-pre EVs (10^10^ particles/ml). Extracellular vesicles-free serum was used in all the above complete media. After incubation for 24 h, analysis was performed using the Annexin V-FITC/PI Apoptosis Detection Kit (Vazyme) following the manufacturer’s instructions. Apoptosis rate of each group was determined using cell flow cytometry.

For further analysis, TUNEL BrightGreen Apoptosis Detection Kit (Vazyme) was used to detect chondrocyte apoptosis. Nuclei of apoptotic cells emitted green fluorescence under the excitation light of the fluorescence microscope. Apoptosis of each group was observed using a fluorescence microscope and photographed.

### Migration analysis of chondrocytes

To explore the effects of EVs and LPS-pre EVs on chondrocyte migration, scratch-wound assay and transwell assay were conducted. After digestion, chondrocytes were randomly divided into four groups including (1) control group, (2) IL-1β + PBS group, (3) IL-1β + EVs group, and (4) IL-1β + LPS-pre EVs group. Chondrocytes (2 × 10^5^ cells) were seeded on six-well plates and cultivated to obtain 100% confluence. A sterile pipette tip (200 μl) was used to make a scratch on the cell layer. Cells were washed three times with PBS, then medium containing PBS, IL-1β (10 ng/ml) + PBS, IL-1β (10 ng/ml) + EVs (10^10^ particles/ml), or IL-1β (10 ng/ml) + LPS-pre EVs (10^10^ particles/ml) was added to the different groups. Extracellular vesicles-free serum was used in all the above complete media. Photos were taken at 0 h, 24 h, and 48 h. The time span of the results was 48 h to exclude the influence of cell proliferation on the results. Cells were treated with mitomycin (1 μg/ml) for 1 h prior to the experiment. EVs and LPS-pre EVs effects on migration of chondrocytes were analyzed by transwell assay. In summary, chondrocytes (2 × 10^4^cells) were inoculated in the upper layer of 24-well transwell chamber (pore size, 8 μm; Corning), whereas 600 μl complete medium consisting of PBS, IL-1β (10 ng/ml) + PBS, IL-1β (10 ng/ml) + EVs (10^10^particles/ml), or IL-1β (10 ng/ml) + LPS-pre EVs (10^10^ particles/ml) was added to the lower chamber and allowed to incubate for 24 h. After incubation, the upper chamber was fixed with 4% paraformaldehyde for 15 min, followed by staining with 0.5% crystal violet for 30 min, and washed three times with PBS. The upper surface of the upper chamber was swabbed to remove cells that did not move to the lower surface. Cell migration of each group was observed under a microscope, and four fields were picked randomly.

### Western blot analysis

Western blot analysis was conducted following a procedure described previously [45]. Cell lysates or extracellular vesicles were centrifugated to obtain the supernatant. 6X sodium dodecyl sulfate (SDS) loading buffer was added to the supernatant and the mixture was boiled for 10 min. The extracted protein solution was subjected to SDS-polyacrylamide gel electrophoresis (PAGE) and electro-transferred to polyvinylidene fluoride (PVDF) membrane. The samples were incubated with primary and secondary antibodies as described previously [46]. Development and semi-quantitative analysis were then carried out. Primary antibodies used included anti-COL2A1 (1:2000; Abcam; ab188570), anti-aggrecan (1:1500; Proteintech; 13880-1-AP), anti-ADAMTS5 (1:2000; Abcam; ab182975), anti-CD9 (1:2000; Abcam; ab223052), anti-CD63 (1:2000; Abcam; ab134045), anti-CD81 (1:2000; Abcam; ab109201), anti-Alix (1:1500; Proteintech; 12422-1-AP), anti-tubulin (1:2000; Proteintech; 10094-1-AP), anti-TSG101 (1:2000; Proteintech; 14497-1-AP), and anti-Ago2(1:2000; Abcam; ab186733).

### Immunofluorescence staining

Mouse knee samples were decalcified, dehydrated, and waxed before being cut into 5-μm-thick sections for immunofluorescence analysis. After dewaxing with xylene and gradient alcohol, sections were sealed with 10% goat serum at room temperature for 1 h and incubated overnight at 4 °C with different primary antibodies including anti-COL2A1 (1:200; Abcam; ab34712), anti-aggrecan (1:200; Proteintech; 13880-1-AP), and anti-ADAMTS5 (1:200; Abcam; ab182795). Each section was then rinsed there times with PBS, followed by incubation for 1 h with green or red fluorescence under ambient temperature. Sections were washed three times with PBS, and nuclei were stained with DAPI. Staining of each section was observed and recorded using fluorescence microscopy.

In immunofluorescence staining of cells, chondrocytes were inoculated in a 24-well plate. After chondrocytes of each group reached 70–80% confluence in the 24-well plates, they were fixed with 4% paraformaldehyde for 15 min and washed three times with PBS. Chondrocytes were then blocked with 5% goat serum at room temperature for 15 min and incubated overnight at 4 °C with the following primary antibodies: anti-COL2A1 (1:200; Abcam; ab34712), anti-aggrecan (1:200; Proteintech; 13880-1-AP), and anti-ADAMTS5 (1:200; Abcam; ab182795). The same procedure was used for immunofluorescence staining of tissue.

### Transfection of small interfering RNA

Small interfering RNA (siRNA) targeting human ADAMTS5 (siRNA-ADAMTS5) and Ago2 (siRNA-Ago2), and their scrambled control siRNAs (siRNA-NC), were purchased from TSINGKE (Nanjing, China). Cells were transfected using Lipofectamine 2000 reagent (Invitrogen) following the manufacturer’s instructions.

### MiRNA target prediction and protein–protein interaction analysis

STRING (http://string-db.org) was used to construct protein interaction network. TargetScan (http://www.targetscan.org), PicTar (https://pictar.mdc-berlin.de), and miRWalk (http://mirwalk.umm.uni-heidelberg.de) were used to predict miRNA target gene of the protein of interest through intersection of prediction results.

### MiRNA microarray assay

miRNA arrays of EVs and LPS-pre EVs of respective samples were conducted by Suri Medicine Company (Nanjing, China). Fragmentation mixtures were crossed using Agilent-Human microRNA array 21.0. In addition, microarray analysis was performed on Affymetrix (Santa Clara, CA, USA) miRNA 4.0 platform. Samples were labeled and microarrays were hybridized and washed following the specific protocols (Agilent Technologies Inc., Santa Clara, CA, USA).

### Real-time RT-PCR

Total cellular RNA was extracted using TRIzol reagent (Invitrogen, Carlsbad, CA, USA). Purity and content of total RNA were determined using a NanoDrop 2000 spectrophotometer (NanoDrop Technologies, Thermo Scientific, USA). PrimeScript™ RT Master Mix (TaKaRa, Japan) was used to synthesize cDNA, and SYBR Green PCR master mix (Applied Biosystems, Foster City, CA) was used to conduct RT-PCR using a QuantStudio 5 Real-Time PCR System (Applied Biosystems, USA). Target gene expression level was normalized using glyceraldehyde 3-phosphate dehydrogenase (GAPDH) and calculated by the 2^−△△CT^ approach. GAPDH and ADAMTS5, let-7b, and Argonaut-2 (Ago2) specific primers were obtained from RiboBio Co, Ltd. (Guangzhou, China). Primers used were **ADAMTS5** forward 5′-GAACATCGACCAACTCTACTCCG-3′ and reverse 5′-CAATGCCCACCGAACCATCT-3′; **let-7b** forward 5′-GGGTGAGGTAGTAGGTTGTGTG-3′ and reverse 5′-CAGGGAAGGCAGTAGGTTGT-3’; **Ago2** forward 5′-GGCTGCTCACCCAATGTATCAAGA-3′ and reverse 5′-AACCGTTCGTTTTGGCGTTGAT-3′; **GAPDH** forward 5′-GCACCGTCAAGGCTGAGAAC-3′ and reverse 5′-TGGTGAAGACGCCAGTGGA-3′.

### Luciferase reporter assay

Sequences corresponding to the 3′-UTR of ADAMTS5 mRNA and containing the wild-type (WT) or mutated (MUT) let-7b binding sequence were synthesized by GeneScript (Nanjing, China). The sequences were subsequently cloned to XbaI and FseI restriction sites in control pGL3 luciferase reporter vector (Promega, Madison, WI, USA). The constructed pGL3 luciferase control reporter vector was then transfected into 293T tool cells. Expression of fluorescent marker genes in cells was observed under fluorescence microscope 24 h after transfection. Luciferase expression was determined using Dual-Luciferase® Reporter Assay System Kit (E1910, Promega).

### Safranin-O and Fast Green staining

Tissue sections were obtained as described under immunofluorescence staining section. Sections were dewaxed with xylene and gradient alcohol. Staining was performed using Safranin-O and Fast Green FCF Stain Kit (Solarbio) following the manufacturer’s instructions. The sections were dipped in Fast Green dye solution for 5 min and then rinsed with running water for 3 min. Sections were then immersed in Safranin-O dye solution for 5 min and directly dehydrated with gradient alcohol. After dehydration, sections were sealed with resin. Staining of each section was observed under a microscope and photographed.

### Induction of osteoarthritis

Animal experiments were performed following guidelines of the Animal Research Committee of Nanjing Medical University. OA induction was conducted on male C57BL/6 mice (10 weeks) through the right knee medial meniscus DMM. Anterior cruciate ligament and medial meniscus were removed during DMM surgery. The skin and muscle were incised at the operation site by sham operation. Postoperatively, each mouse was treated with buprenorphine (0.05 mg/kg) to relieve pain and gentamicin (5 mg/kg) to prevent infection.

### Intra-articular injection of extracellular vesicles

All mice that underwent DMM surgery were randomly divided into three groups, and mice that received the sham operation were placed in the sham operation group. The groups included (1) sham operation group, (2) PBS group, (3) PBS-EVs group, and (4) PBS-LPS-pre EVs group. Mice in each group received intra-articular injection of PBS (10 μl), PBS-EVs (10 μl, 10^11^ particles/ml), or PBS-LPS-pre EVs (10 μl, 10^11^ particles/ml) twice-weekly, and all animals were allowed to jog on a specific treadmill at 10 m/min for 40 min per day for 6 weeks.

### Intra-articular injection of antagomiR

All mice were randomly divided into two groups: (1) LPS-pre EVs + antagomiR-NC group and (2) LPS-pre EVs + antagomiR-let-7b group. The first and second groups received antagomiR-NC (10 μl, 200 nmol/ml) or antagomiR-let-7b (10 μl, 200 nmol/ml) through intra-articular injections twice per week for 3 weeks, respectively. All groups of mice underwent DMM surgery. After surgery, the first and second groups received intra-articular injections of LPS-pre EVs (10 μl, 10^11^ particles/ml) + antagomiR-NC (10 μl, 200 nmol/ml) or LPS-pre EVs (10 μl, 10^11^ particles/ml) + antagomiR-let-7b (10 μl, 200 nmol/ml) twice-weekly, respectively, for 6 weeks. All mice were allowed to jog on a specific treadmill at a pace of 10 m/min for 40 min per day for 6 weeks.

### Statistical analysis

GraphPad Prism 8.0 (GraphPad Software, Inc., San Diego, CA) and SPSS 17.0 (IBM, Armonk, NY, USA) were used for statistical analysis. Data were presented as mean ± SD. Student’s *t* test was adopted to compare two groups, whereas one-way analysis of variance (ANOVA) followed by Bonferroni post hoc test was used for comparisons among several groups. *P* < 0.05 indicated statistical significance.

## Results

### Identification of SMSCs and LPS-pre SMSCs

**Fig. 1:**
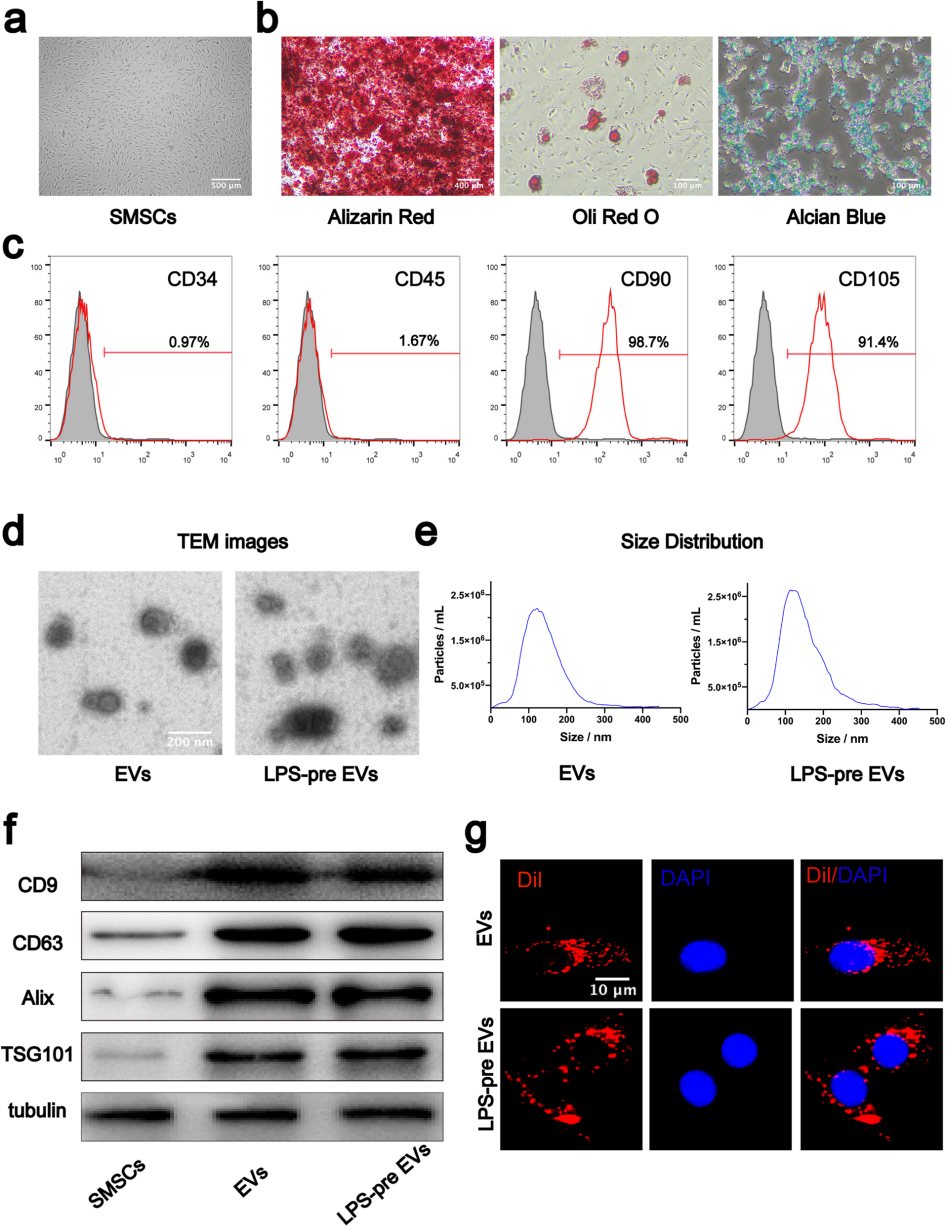
Identification of human SMSCs and extracellular vesicles. **a** SMSCs exhibited a spindle-like morphology. **b** SMSCs showed multidirectional differentiation potential of osteogenesis, adipogenesis, and chondrogenesis. **c** Characteristic cell surface markers of SMSCs were analyzed by flow cytometry. **d** EVs and LPS-pre EVs observed through TEM. **e** Particle size distribution of EVs and LPS-pre EVs analyzed by NanoSight. **f** extracellular vesicles markers (CD9, CD63, Alix, TSG101) analyzed by western blot. **g** Chondrocyte internalization of EVs and LPS-pre EVs as observed under fluorescence microscope

SMSCs were extracted from knee joint synovial tissues, and those in passage 5 were selected for further analysis. After reaching 80–90% confluence, cells displayed typical MSC morphology with spindle-like shape (Fig. 1a). SMSCs were cultured in osteogenic, adipogenic, or chondrogenic medium. Alizarin Red staining, Oli Red O staining, and Alcian Blue staining were used to explore differentiation of SMSCs during osteogenesis (Fig. 1b left panel), lipogenesis (Fig. 1b middle panel), and chondrogenesis (Fig. 1b right panels), respectively. Analysis of surface antigen expression using flow cytometry showed that SMSCs were positive for CD90 and CD105 and negative for CD34 and CD45 (Fig. 1c).

### Isolation and identification of extracellular vesicles derived from SMSCs and LPS-pre SMSCs

Extracellular vesicles were extracted from the supernatant medium of SMSCs with or without LPS. Morphology of EVs and LPS-pre EVs was observed under a transmission electron microscope (TEM). Morphological analysis showed that both EVs and LPS-pre EVs were round or disk-shaped (Fig. 1d). Diameters of EVs and LPS-pre EVs were determined using NanoSight. Most EVs and LPS-pre EVs had a size ranging between 50 and 200 nm (Fig. 1e). Western blot results showed presence of markers of EVs including CD9, CD63, Alix, and TSG101 in EVs and LPS-pre EVs but not in SMSCs (Fig.1f).

### Internalization of extracellular vesicles by chondrocytes

To explore whether chondrocytes can internalize extracellular vesicles, Dil dye was used to label EVs and LPS-pre EVs. The labeled EVs and LPS-pre EVs were co-incubated with chondrocytes for 24 h and washed three times with PBS. DAPI stain was used to stain nuclei, and samples were observed under a fluorescence microscope. Analysis showed that chondrocytes effectively internalized both EVs and LPS-pre EVs (Fig. 1 g).

### LPS-pre EVs is superior to EVs in promoting chondrocytes proliferation and migration and in inhibiting cell apoptosis

**Fig. 2:**
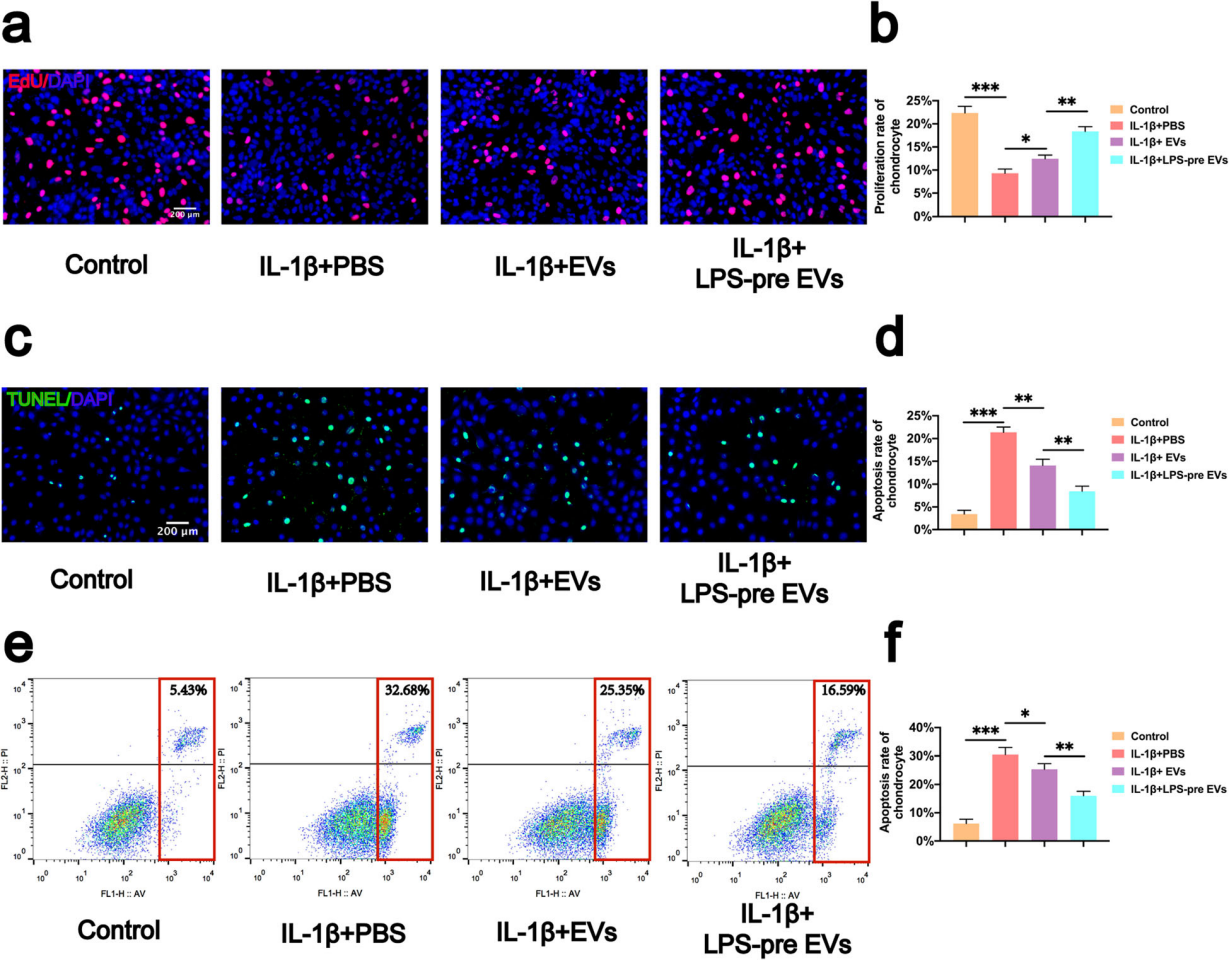
Effects of LPS-pre EVs on proliferation and apoptosis of chondrocytes. **a**, **b** Proliferation rate of chondrocytes was determined by EdU assay (*n* = 3, one-way ANOVA). **c**, **d** Apoptosis rate of chondrocytes was determined by TUNEL assay (*n* = 3, one-way ANOVA). **e**, **f** Apoptosis rate of chondrocytes was analyzed by flow cytometry (*n* = 3, one-way ANOVA). **b**, **d**, **f** Values are presented as mean ± SD. *P < 0.05, **P < 0.01, ***P < 0.001, one-way ANOVA

**Fig. 3:**
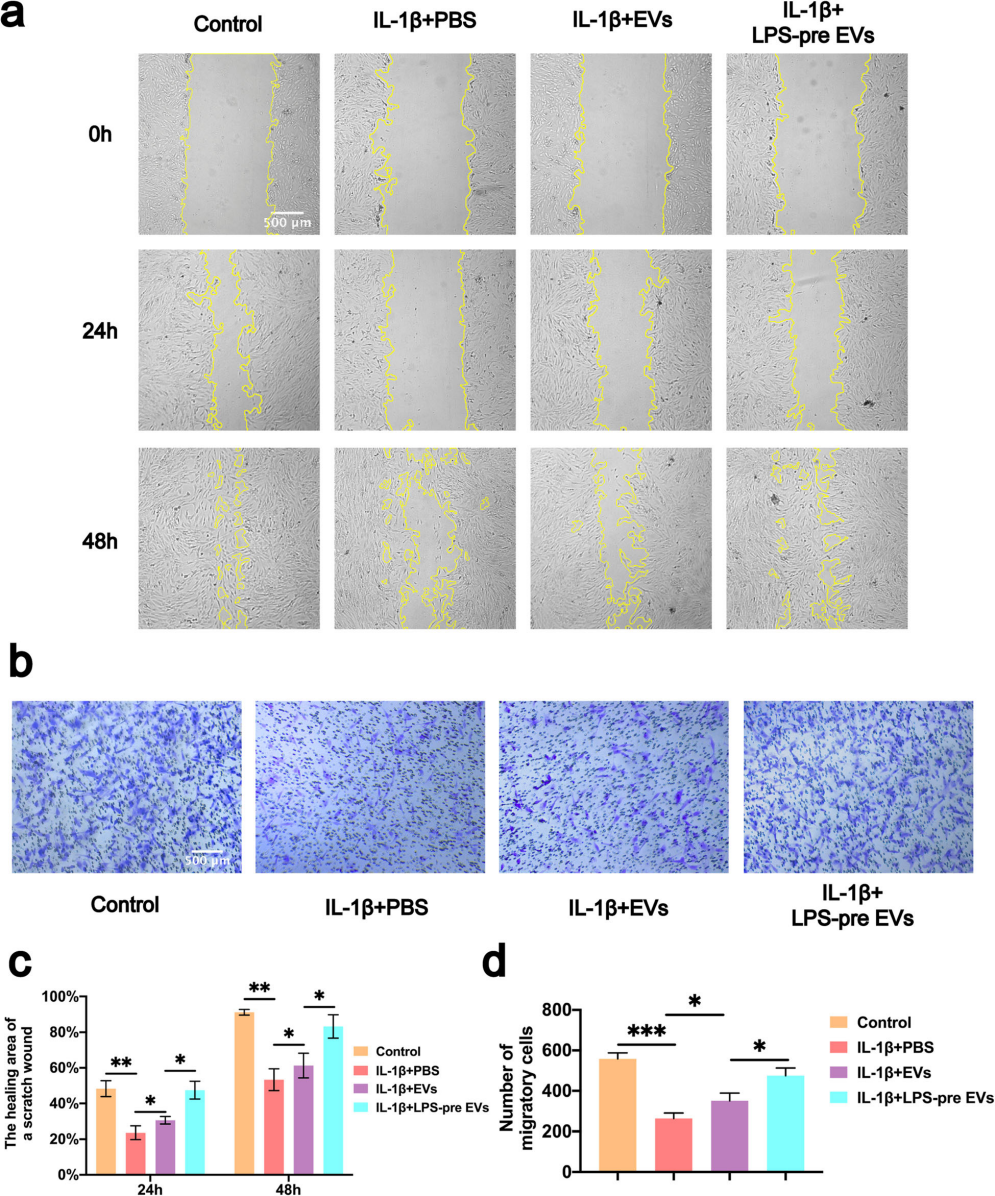
Effects of LPS-pre EVs on migration of chondrocytes. **a**, **c** Images of scratch assays on chondrocytes obtained under a light microscope (*n* = 3, one-way ANOVA). **b**, **d** Migration of chondrocytes as verified by transwell assay. Crystal violet was used to stain migrating chondrocytes (*n* = 3, one-way ANOVA). **c**, **d** Values are presented as mean ± SD. *P < 0.05, **P < 0.01, ***P < 0.001, one-way ANOVA

To compare the effects of LPS-pre EVs and EVs on proliferation, migration, and apoptosis inhibition of chondrocytes, experiments were conducted with LPS-pre EVs and EVs. Proliferation of chondrocytes was explored using EdU assays (Fig. 2a, b). The results of the EdU assay showed that the number of EdU-stained chondrocytes was significantly higher in both EVs and LPS-pre EVs groups compared with the number in the PBS group (Fig. 2a). However, treatment with LPS-pre EVs showed significantly higher number of EdU-stained cells compared with treatment with EVs. Inhibitory effect of LPS-pre EVs on chondrocyte apoptosis rate was determined using the TUNEL assay (Fig. 2c, d) and flow cytometry analysis (Fig. 2e, f). Although both EVs and LPS-pre EVs inhibited chondrocyte apoptosis caused by IL-1β, the effect of LPS-pre EVs in inhibiting chondrocyte apoptosis was significantly higher compared with that of EVs (Fig. 2c). Notably, both EVs and LPS-pre EVs had higher inhibition on chondrocyte apoptosis compared with the PBS group. Similar results were obtained using flow cytometry analysis of apoptotic cells (Fig. 2e, f). The effect of the two types of extracellular vesicles on chondrocyte migration was explored using scratch assays (Fig. 3a, c) and transwell assay (Fig. 3b, d). The analysis showed that EVs and LPS-pre EVs significantly increased migration of chondrocytes compared with the PBS group. However, LPS-pre EVs treatment showed significant increase in the migration capability of chondrocytes compared with EVs alone.

### LPS-pre EVs inhibited decrease of aggrecan and COL2A1 and increase of ADAMTS5 caused by IL-1β

**Fig. 4:**
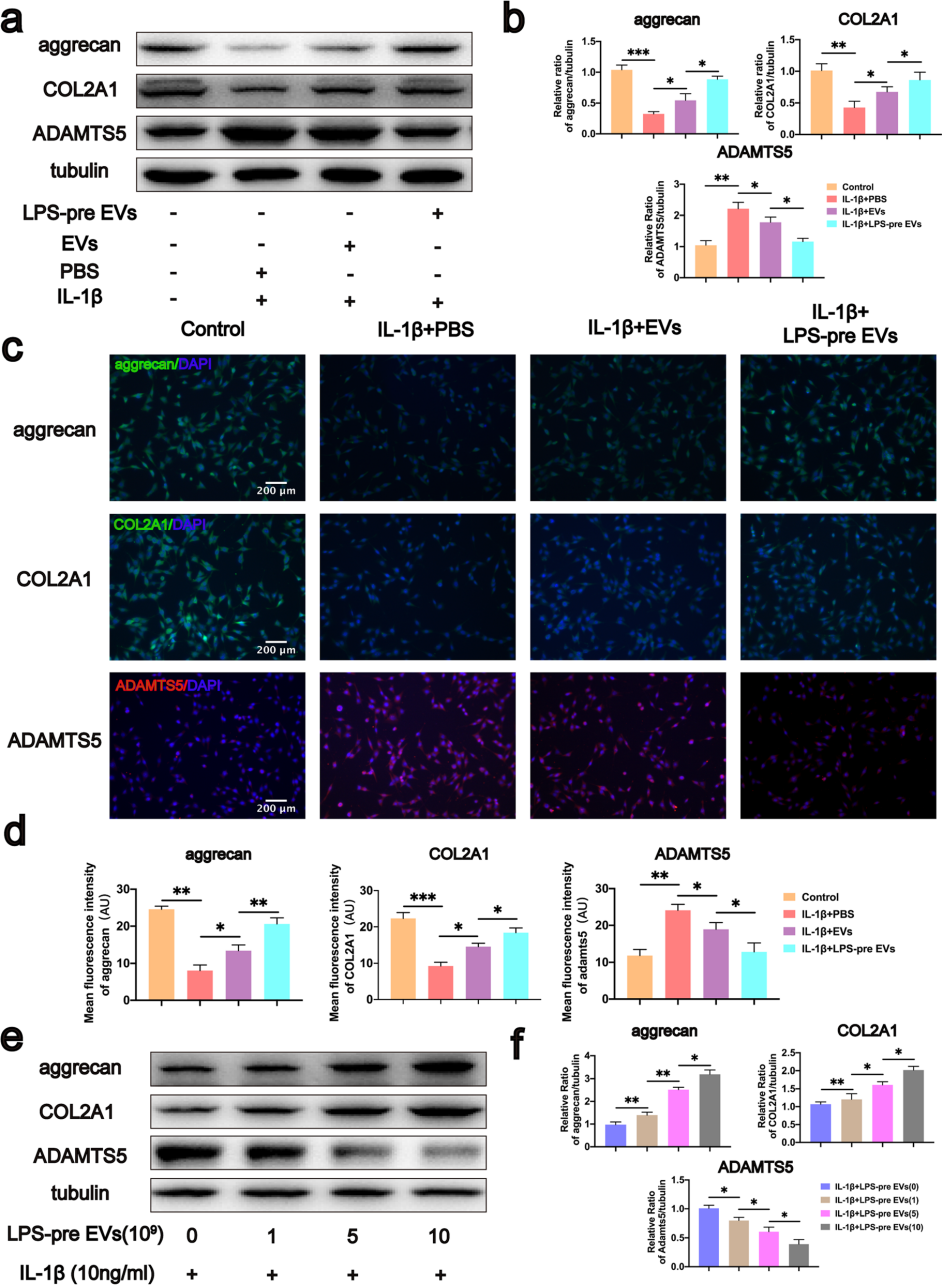
Effects of LPS-pre EVs on protein expression levels in chondrocytes. **a**, **b** Protein expression of aggrecan, COL2A1, and ADAMTS5 in chondrocytes of each group analyzed by western blot (*n* = 3, one-way ANOVA). **c**, **d** Protein expression of aggrecan, COL2A1, and ADAMTS5 in chondrocytes of each group analyzed by immunofluorescence staining (*n* = 3, one-way ANOVA). **e**, **f** Protein expression of aggrecan, COL2A1, and ADAMTS5 in chondrocytes treated with different concentrations of LPS-pre EVs and analyzed by western blot (*n* = 3, one-way ANOVA). **b**, **d**, **f** Values are presented as mean ± SD. *P < 0.05, **P < 0.01, ***P < 0.001, one-way ANOVA

Chondrocytes underwent analysis to evaluate the effect of LPS-pre EVs on regulating chondrocyte protein expression. Western blot analyses were used to determine expression levels of aggrecan and COL2A1, the most important components in ECM. Analysis showed that LPS-pre EVs significantly inhibited decrease of aggrecan and COL2A1 caused by IL-1β in chondrocytes compared with the levels in the EVs and PBS group (Fig.4a, b). Protein interaction predicted by STRING (https://string-db.org) and reported in previous studies showed that ADAMTS5 is closely related to aggrecan and COL2A1 [47, 48]. In addition, ADAMTS5 plays an important role in the degradation process of ECM. Therefore, protein expression level of ADAMTS5 of each group was determined. Analysis showed that both EVs and LPS-pre EVs inhibited expression of ADAMTS5 compared with the PBS group. Notably, the effect of LPS-pre EVs on the inhibiting ADAMTS5 expression was significantly higher compared with that of EVs. To verify this result, cell immunofluorescence was performed directly on chondrocytes in each group, and similar results were observed using immunofluorescence analysis (Fig. 4c, d). To explore the dose–effect relationship of LPS-pre EVs, different concentrations of LPS-pre EVs were used to treat chondrocytes. Protein expression levels in chondrocytes were then determined by western blot analyses. Analysis showed that a concentration of LPS-pre EVs of 10^10^ particles/ml had the highest inhibition effect on decreasing aggrecan and COL2A1 levels and increasing ADAMTS5 level (Fig. 4e, f). These findings show that LPS-pre EVs has significantly higher effect in inhibiting decrease in aggrecan and COL2A1 levels and increasing of ADAMTS5 level compared with EVs.

### LPS-pre EVs significantly inhibited cartilage destruction in DMM-induced mouse model of OA

**Fig. 5:**
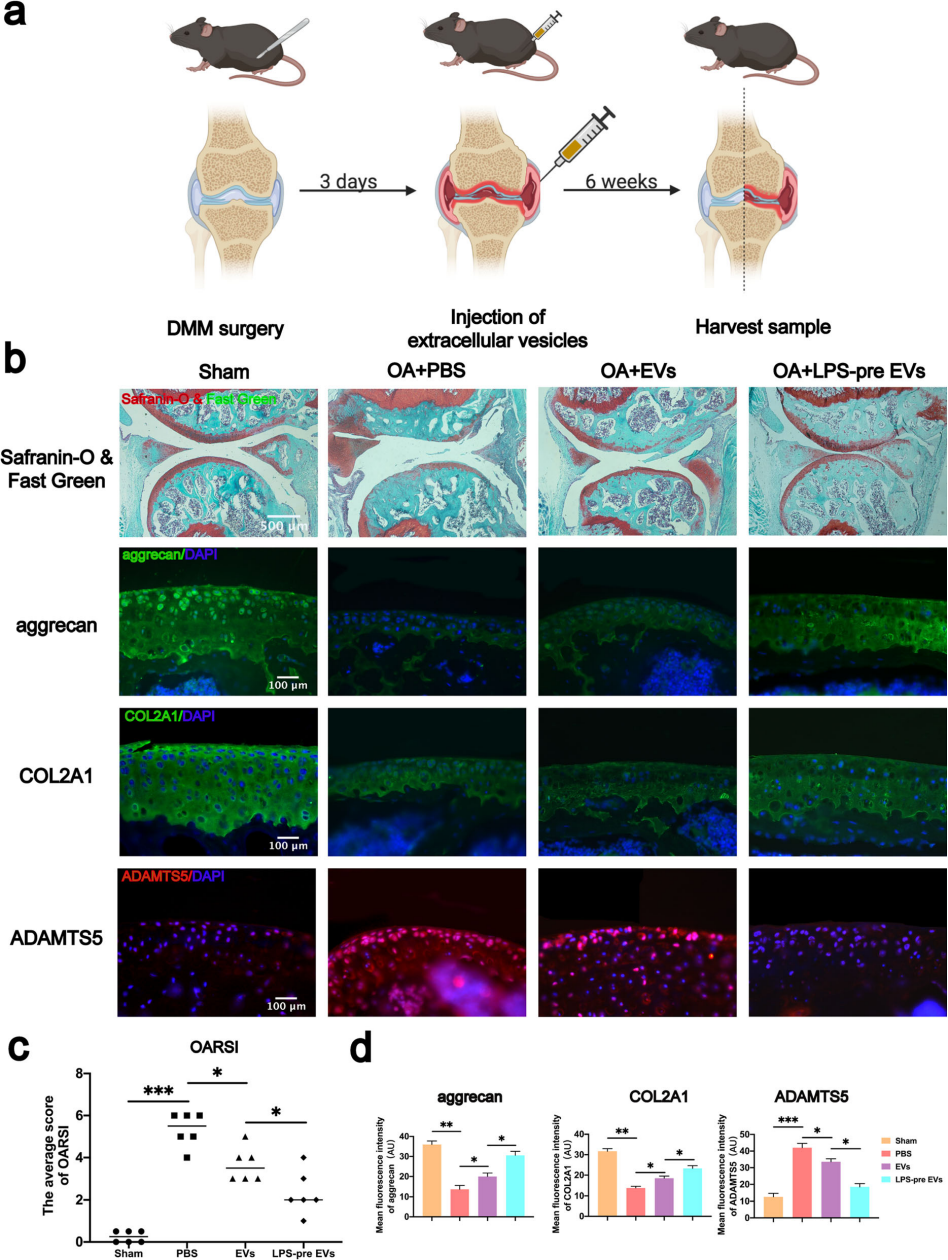
Effect of LPS-pre EVs on cartilage protection and protein expression in vivo. **a** Animal experiment model diagram. **b** Safranin-O and Fast Green and immunofluorescence staining of each group of mouse knee section (*n* = 6, one-way ANOVA). **c** OARSI scores in each group (*n* = 6, one-way ANOVA). **d** Protein immunofluorescence intensity of aggrecan, COL2A1, and ADAMTS5 in each group (*n* = 6, one-way ANOVA). **c**, **d** Values are presented as mean ± SD. *P < 0.05, **P < 0.01, ***P < 0.001, one-way ANOVA

To explore the role of LPS-pre EVs in protecting articular cartilage, the DMM-mediated mouse model of OA was established. All DMM-induced mouse models of OA received different treatments as described in the methods section (Fig. 5a). Safranin-O and Fast Green staining were performed on the sample sections of each group. EVs group and LPS-pre EVs group showed a protective effect on articular cartilage tissue (Fig. 5b). The cartilage surface in the LPS-pre EVs group was smoother, the subchondral bone was not exposed, and the cartilage layer was thicker compared with the PBS group and the EVs group. These findings indicate that LPS-pre EVs reduced cartilage damage in the mouse model and had significant protective effect on cartilage compared with the EVs group (Fig.5b). In addition, tissue immunofluorescence technology was used to determine protein expression sections from each group. Analysis showed that LPS-pre EVs significantly reduced ADAMTS5 expression and increased expression levels of aggrecan and COL2A1 in the tissue (Fig. 5b, d). This finding was consistent with the results obtained from western blot analysis. Osteoarthritis Research Society International (OARSI) scoring was used on mouse samples of each group. The results showed that, LPS-pre EVs group significantly reduced the OARSI score compared with the other groups (Fig. 5c). These findings show that LPS-pre EVs have better cartilage protection compared with EVs in the mouse OA model.

### LPS-pre EVs regulates protein expression levels through miRNA

**Fig. 6:**
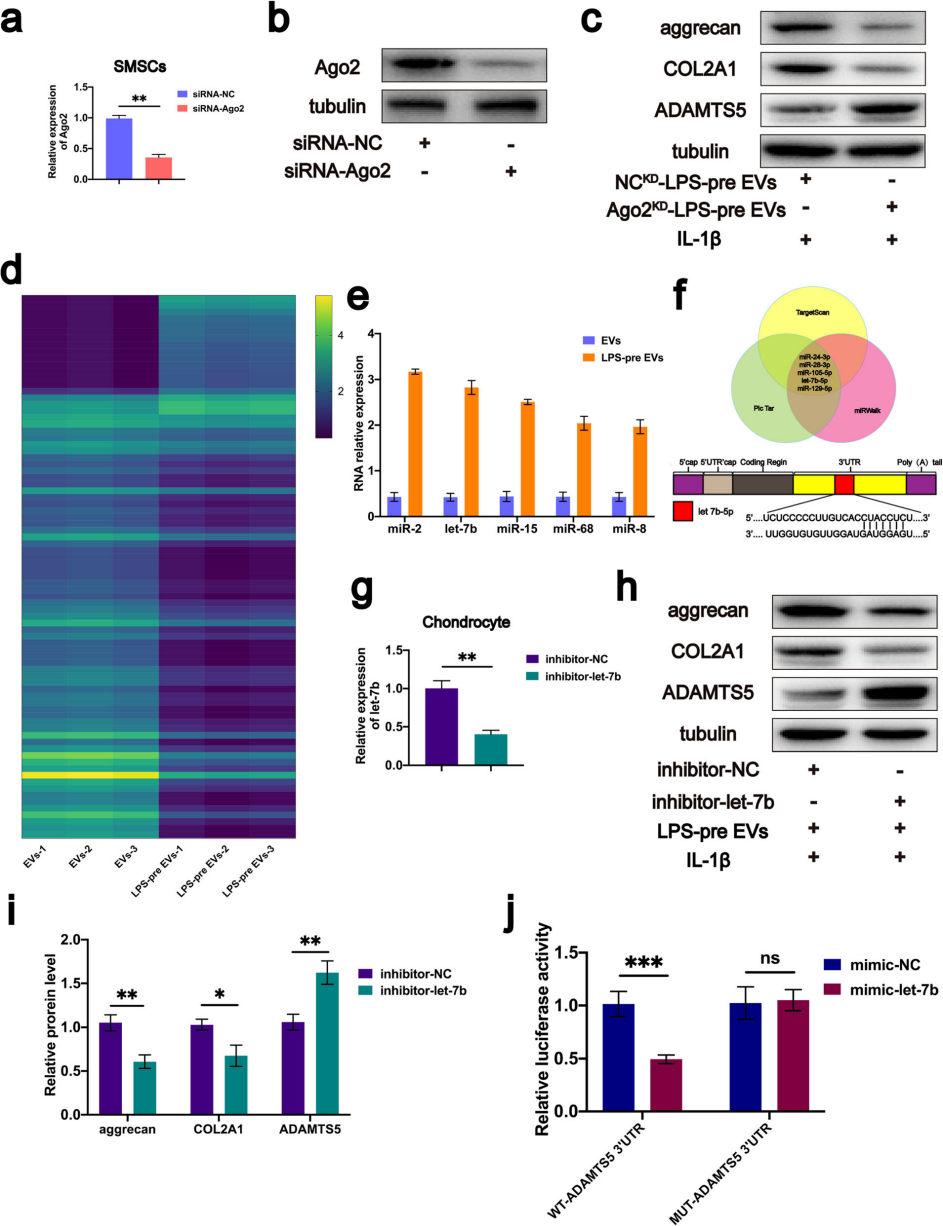
let-7b is upregulated in LPS-pre EVs, and knockdown of let-7b inhibits LPS-pre EVs-mediated effects on protein expression in vitro. **a** SMSCs were transfected with siRNA-Ago2, and transfection efficiency was evaluated using real-time PCR (*n* = 6, Student’s *t* test). **b** Protein expression of Ago-2 analyzed by western blot (*n* = 3, one-way ANOVA). **c** Protein expression of aggrecan, COL2A1, and ADAMTS5 in chondrocytes treated with NC^KD^-LPS-pre EVs or Ago2^KD^-LPS-pre EVs. **d** Heat map of miRNA levels between EVs and LPS-pre EVs groups (*n* = 3, Student’s *t* test). **e** Comparisons of the five most significantly upregulated miRNAs between EVs and LPS-pre EVs. **f** Venn diagram showing the five most significant targets for ADAMTS5 identified by three independent miRNA target prediction programs, and the target sequence of let-7b estimated within 3′-UTR of ADAMTS5. **g** Inhibitor-let-7b was transfected into chondrocytes, and RT-PCR was performed to evaluate transfection efficiency (*n* = 6, Student’s *t* test). **h**–**i** Aggrecan, COL2A1, and ADAMTS5 protein expression levels within chondrocytes after let-7b knockdown (*n* = 3, Student’s *t* test). **j** Luciferase reporter assay showing that ADAMTS5 is the target gene for let-7b (*n* = 3, Student’s *t* test). **a**, **g**, **i**, **j** Values are presented as mean ± SD. NS, no significance; *P < 0.05, **P < 0.01, ***P < 0.001, Student’s *t* test and one-way ANOVA

Previous studies report that miRNA is a vital component of extracellular vesicles, by playing roles in cell function modulation and cell communication [49]. In addition, Ago2 an RNA-induced silencing complex component is a critical miRNA regulator by regulating miRNA-mediated mRNA cleavage activity or by inhibiting activity at the translational level [50]. Ago2 protein is localized in EVs [51, 52]. The biological function of LPS-pre EVs may be correlated with miRNA in extracellular vesicles. Therefore, Ago2 was knocked down in SMSCs using siRNA-Ago2 and the transfection efficiency was determined by real-time RT-PCR (Fig. 6a). Moreover, western blot analysis was used to verify the protein expression level of Ago2. Western blot analysis results showed that the protein expression level of Ago2 was significantly reduced after Ago2 knocked down (Fig. 6b). SMSCs with Ago2 knockdown (Ago2^KD^-SMSCs) were then cultured in medium containing LPS, and the supernatant was obtained to extract extracellular vesicles with Ago2 knockdown (Ago2^KD^-LPS-pre EVs). Ago2^KD^-LPS-pre EVs and NC^KD^-LPS-pre EVs were added to the chondrocyte medium, separately. After 72 h, chondrocyte proteins in each group were extracted and analyzed using western blot analysis. Western blot analysis showed that Ago2^KD^-LPS-pre EVs did not reduce expression of ADAMTS5 or reduce degradation of COL2A1 and aggrecan (Fig. 6c). This finding implied that miRNAs contained in LPS-pre EVs, may exert a biological function and eventually contribute to inhibition of ECM degradation.

### Let-7b may be a key miRNA in the regulation of protein expression level by LPS-pre EVs

Expression level of aggrecan and COL2A1 can be determined by the ADAMTS5 expression level as mentioned above. We speculated that there may be a specific miRNA in LPS-pre EVs that specifically binds to ADAMTS5 mRNA, thus reducing ADAMTS5 expression. Therefore, total RNA was extracted from in EVs and LPS-pre EVs and microarray analysis performed. miRNA microarray analysis (Fig. 6d) showed 18 miRNAs upregulated and 64 downregulated in the LPS-pre EVs group compared with the EVs group. The top five upregulated miRNAs including miR-2, let-7b, miR-15, miR-68, and miR-8 were selected based on the miRNA profile data (Fig.6c). miR-2, miR-15, miR-68, and miR-8 are novel miRNAs. In addition, all miRNA that target ADAMTS5 were predicted using three online databases including TargetScan, PicTar, and DIANA. The intersection of the three prediction results was used and five miRNA sequences of miR-24-3p, miR-28-3p, miR-105-5P, let-7b-5p, and miR-129-5p were identified (Fig. 6f). Analysis showed that let-7b was the only miRNA that was highly expressed in LPS-pre EVs and within the predicted miRNA range (Fig.6e, f). Therefore, let-7b may specifically bind to mRNA of ADAMTS5, thus inhibiting expression of ADAMTS5.

### Knockdown of let-7b inhibits regulation of LPS-pre EVs on protein expression

**Fig. 7:**
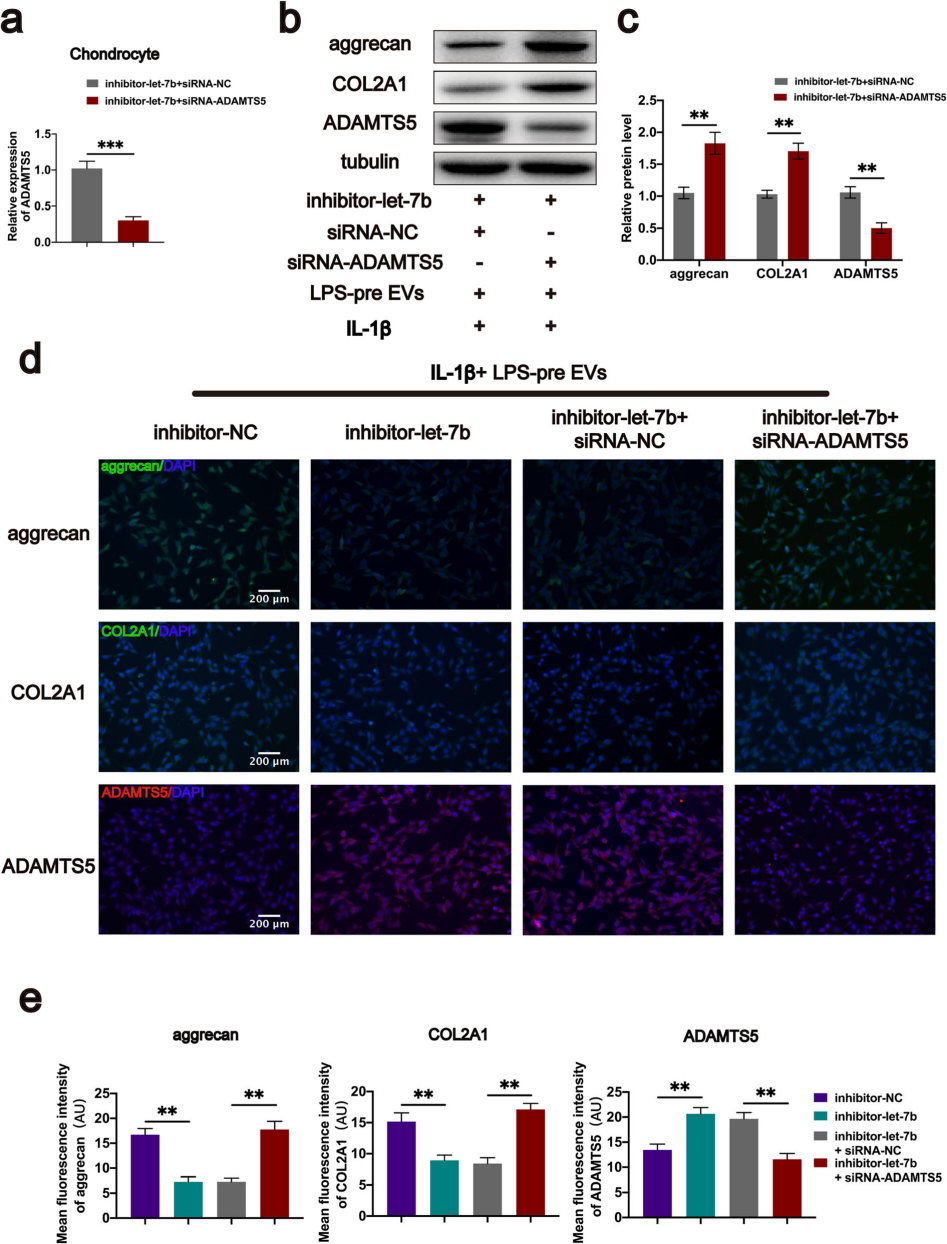
Let-7b promotes aggrecan expression by targeting ADAMTS5 in vitro. **a** Chondrocytes with let-7b knockdown were transfected with siRNA-ADAMTS5, and transfection efficiency was evaluated by real-time PCR (*n* = 6, Student’s *t* test). **b**, **c** Protein expression of aggrecan, COL2A1, and ADAMTS5 in chondrocytes after knocking down ADAMTS5 (*n* = 3, Student’s *t* test). **d**, **e** Protein immunofluorescence intensity of aggrecan, COL2A1, and ADAMTS5 in each group (*n* = 3, one-way ANOVA). **a**, **c**, **e** Values are presented as mean ± SD. **P < 0.01, ***P < 0.001, Student’s *t* test and one-way ANOVA

Let-7b was knocked down on chondrocytes and real-time RT-PCR was used to verify the knockdown efficiency (Fig. 6 g). The two groups of cells were treated with IL-1β and LPS-pre EVs, and the proteins of each group were extracted for western blot analysis. Western blot analysis results showed that LPS-pre EVs could not play its role in inhibiting decrease of aggrecan and COL2A1 and increase of ADAMTS5 after let-7b was knocked down in chondrocytes (Fig. 6 h, i). Immunofluorescence staining analysis of cells in both groups showed consistent results with those of western blot analyses (Fig. 7d, e).

### Let-7b regulates ADAMTS5 by directly targeting the 3′- UTR

To explore whether the 3′-UTR of ADAMTS5 served as the direct target for let-7b, luciferase reporter assay was carried out on 293 T cells. Analysis showed that co-transfection of ADAMTS5 WT (rather than MUT) luciferase construct with let-7b mimic reduced luciferase activity (Fig. 6j). This finding shows that ADAMTS5 is the target gene of let-7b.

### Let-7b inhibits degradation of aggrecan and COL2A1 by targeting ADAMTS5

To further explore the relationship between let-7b and ADAMTS5, chondrocytes with let-7b knockdown were transfected with siRNA-ADAMTS5. Transfection efficiency was determined by real-time RT-PCR (Fig. 7a). After 72 h of transfection, cell proteins of each group were extracted for western blot analysis. ADAMTS5 protein expression level was significantly reduced and the aggrecan and COL2A1 protein expression levels were significantly increased in the let-7b and ADAMTS5 knockdown group compared with the let-7b knockdown group (Fig. 7b, c). Further, immunofluorescence staining of cells in both groups showed consistent results with those from western blot analyses (Fig. 7d, e). These findings show that let-7b inhibits degradation of aggrecan and COL2A1 by targeting ADAMTS5.

### Cartilage protective and protein regulation effects by LPS-pre EVs is inhibited by depletion of let-7b in vivo

**Fig. 8:**
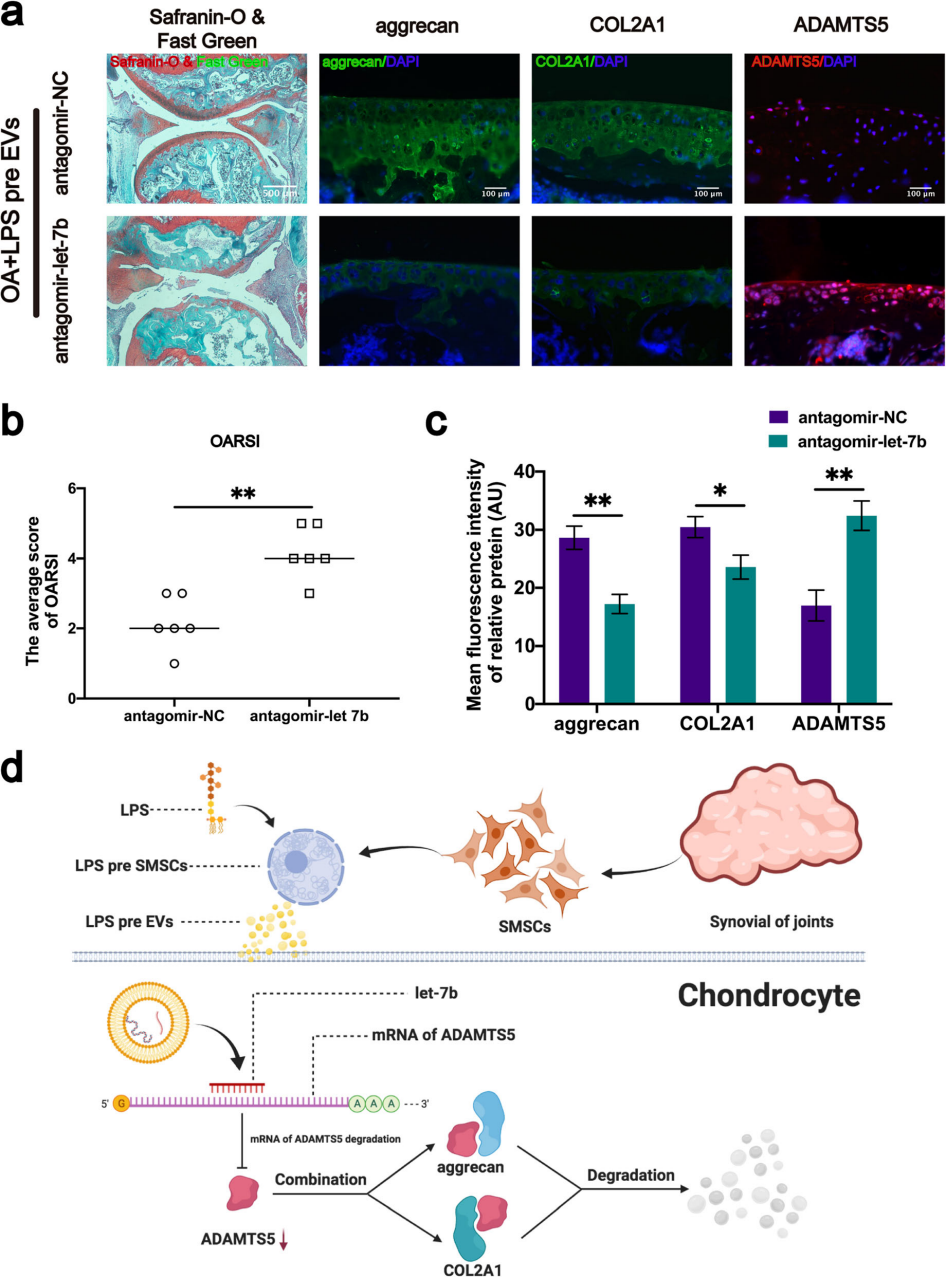
Let-7b promotes aggrecan expression by targeting ADAMTS5 in vivo. **a** Safranin-O, Fast Green, and immunofluorescence staining of each group of mouse knee section. **b** OARSI scores for each group (*n* = 6, Student’s *t* test). **c** Protein immunofluorescence intensity of aggrecan, COL2A1, and ADAMTS5 in each group (*n* = 6, Student’s *t* test). **d** let-7b in LPS-pre EVs directly targets and binds ADAMTS5, inhibits ADAMTS5 expression, and thus inhibits aggrecan and COL2A1 degradation. **b**, **c** Values are presented as mean ± SD. NS, *P < 0.05, **P < 0.01, Student’s *t* test

To explore whether let-7b is important and is involved in LPS-pre EVs-mediated cartilage protection in vivo, antagomiR-let-7b was used in DMM mice model. Sample sections were stained with Safranin-O and Fast Green. Analysis showed that the cartilage of the mice with let-7b knockdown was severely damaged (Fig. 8a), and the OARSI score was significantly higher compared with the group without let-7b knockdown (Fig. 8b). Immunofluorescence staining of the sample sections showed that LPS-pre EVs did not inhibit expression of ADAMTS5 and did not inhibit degradation of aggrecan and COL2A1 in the let-7b knockdown mouse model of OA (Fig. 8a, c). These findings indicate that LPS-pre EVs inhibits expression of ADAMTS5 and degradation of aggrecan and COL2A1 and reduces OARSI score of mouse model of OA through the let-7b.

## Discussion

Inflammation is a key driver of OA pathogenesis [53]. Pro-inflammatory cytokines are significantly elevated in the synovial fluid of OA patients, and they play critical roles in pathogenesis of OA [54, 55]. IL-1β is the most involved proinflammatory cytokines in the catabolic process of OA [55, 56]. Pro-inflammatory IL-1β contributes to OA development by inducing catabolic and destructive processes. IL-1β is overexpressed in the synovial tissue, cartilage, and subchondral bone layer, and induces matrix destructive enzymes in OA [56–59]. Increase in expression levels of these degradative enzymes shift the balance of homoeostasis towards catabolic metabolism, resulting in cartilage degradation. Established mechanisms that prevent OA development mainly depend on inhibition of cartilage degradative enzymes [47, 58, 60]. Therefore, it is important to modulate the inflammatory process to prevent and attenuate OA development.

Recent studies report that MSCs transplantation has a good therapeutic effect on some degenerative diseases [15, 16]. However, direct use of mesenchymal stem cells is limited by possible side effects such as chromosomal variations and immune rejection [23, 24]. Moreover, studies report that mesenchymal stem cells exert their biological functions mainly by activation of resident cells through paracrine mechanisms [61]. Direct use of extracellular vesicles does not cause side effects such as chromosomal variations and immune rejection [27, 28]. Therefore, the use of MSCs-derived extracellular vesicles in the treatment of some degenerative diseases is an excellent treatment strategy. SMSCs have the advantages of easy isolation, high proliferation ability, and slow aging compared with other MSCs [22]. Moreover, SMSCs have the advantage of tissue specificity of cartilage regeneration, making SMSCs have higher potential in cartilage regeneration research [21].

Pretreatment of MSCs with LPS has become a promising approach to treat tissue damage and inflammatory disorders. Several studies report that pretreatment with LPS significantly promotes paracrine protection, and MSCs repair and regeneration capacities [62, 63]. Bone-marrow-derived MSCs can produce excessive extracellular vesicles for exchange of biochemical information between nearby cells, thus maintaining homeostasis and dynamics of the tissue-repair microenvironment [64]. Therefore, LPS pretreatment of extracellular vesicles secreted by SMACs may have a better therapeutic effect on OA.

In this study, LPS-pre EVs secreted by LPS-pre SMSCs were extracted and used in several experiments. LPS-pre EVs significantly enhanced chondrocyte migration and proliferation, and suppressed apoptosis of chondrocytes in vitro, compared with EVs. In addition, LPS-pre EVs inhibited degradation of aggrecan and COL2A1 and increase of ADAMTS5 expression induced by IL-1β. LPS-pre EVs were used in the mouse model of OA. Progression of early-stage OA was delayed, and knee joint cartilage damage caused by OA was prevented by LPS-pre EVs in vivo.

Notably, LPS-pre EVs inhibited degradation of aggrecan and COL2A1 and inhibited increase of ADAMTS5 expression caused by IL-1β, therefore to explore the specific mechanism that the role of miRNAs in extracellular vesicles was determined. The content and type of miRNAs in extracellular vesicles secreted by SMSCs may have been changed after LPS pretreatment, resulting to this observation. A series of experiments were conducted to that LPS inhibits expression of AMAMTS5 protein through let-7b present in LPS, which further inhibits degradation of aggrecan and COL2A1, important components of ECM. In addition, we hypothesized that the mechanism by which LPS-pre EVs promote chondrocyte proliferation and migration and inhibit chondrocyte apoptosis may be related to the high expression of miRNAs. However, due to lack of resources, the mechanism by which LPS-pre EVs promoted chondrocyte proliferation and migration and inhibited chondrocyte apoptosis was not explored in this study. Further studies should explore this mechanism. Regenerative effect of LPS-pre EVs was evaluated using a DMM mouse model. The surgical model of DMM is used as a gold standard for studying OA progression in vivo and provides good reproducibility and a slower disease progression. Analysis showed that LPS-pre EVs effectively prevented progression of cartilage damage in an OA model. Although this study shows that LPS-pre EVs are effective in preventing OA progression, the mechanisms of their therapeutic effects on OA were not fully determined. Therefore, further detailed investigation of the internal contents and mechanism of action of the LPS-pre EVs should be conducted. Furthermore, the effective dose of extracellular vesicles should be explored using animal experiments.

In summary, the findings of this study show that LPS-pre EVs effectively protect cartilage from degeneration and attenuate OA progression. In addition, LPS-pre EVs promoted chondrocyte proliferation and migration, inhibited chondrocyte apoptosis, and modulated expression levels of related proteins. Therefore, LPS-pre EVs are potential targets for treatment of OA.

## Conclusion

The findings of this study showed that LPS-pre EVs promote chondrocyte proliferation, migration, and inhibit chondrocyte apoptosis. Moreover, let-7b contained in LPS-pre EVs directly targets ADAMTS5 and inhibits expression of ADAMTS5 to inhibit degradation of aggrecan and COL2A1. In the mouse model of OA, LPS-pre EVs significantly improved cartilage damage caused by DMM surgery and prevented OA from occurring.

## Data Availability

All relevant data and materials are available from the authors upon reasonable request.

## Abbreviations

LPS: Lipopolysaccharide
EVs: Extracellular vesicles secreted by synovial mesenchymal stem cells
OA: Osteoarthritis
DMM: Destabilization of the medial meniscus
COL2A1: Collagen type II alpha 1
ECM: Extracellular matrix
PBS: Phosphate buffer saline
MSCs: Mesenchymal stem cells
SMSCs: Synovial mesenchymal stem cells
miRNAs: microRNAs
LPS-pre EVs: Extracellular vesicles derived from LPS-preconditioned synovial mesenchymal stem cells
siRNA: Small interfering RNA
ADAMTS5: A disintegrin-like and metalloproteinase domain with thrombospondin-1 motifs 5
THA: Total knee arthroplasty
DMEM/F12: Dulbecco’s Modified Eagle Medium/Nutrient Mixture F-12
FBS: Fetal bovine serum
TEM: Transmission electron microscope
DAPI: 4′,6-Diamidino-2-phenylindole
ANOVA: Analysis of variance
OARSI: Osteoarthritis Research Society International
